# Novel avian paramyxovirus isolated from gulls in Caspian seashore in Kazakhstan

**DOI:** 10.1371/journal.pone.0190339

**Published:** 2017-12-28

**Authors:** Kobey Karamendin, Aidyn Kydyrmanov, Yermukhammet Kasymbekov, Saule Asanova, Klara Daulbayeva, Aigerim Seidalina, Elizaveta Khan, Sally M. Harrison, Ian M. Carr, Simon J. Goodman, Alibek Moldakozhayev, Marat Sayatov

**Affiliations:** 1 Laboratory of Viral Ecology, Institute of Microbiology and Virology, Almaty, Kazakhstan; 2 School of Medicine, Faculty of Medicine and Health, University of Leeds, St. James’s University Hospital, Leeds, United Kingdom; 3 School of Biology, Faculty of Biological Sciences, University of Leeds, Leeds, United Kingdom; University of Maryland at College Park, UNITED STATES

## Abstract

Three isolates APMV/gull/Kazakhstan/5976/2014, APMV/gull/Kazakhstan/ 5977/2014 and APMV/gull/Kazakhstan/5979/2014, were obtained from independent samples during annual surveillance for avian influenza and paramyxoviruses in wild birds from the Caspian Sea coast in Western Kazakhstan, and were initially identified as putative paramyxoviruses on the basis of electron microscopy. Hemagglutination Inhibition Assays with antisera to nine known APMV serotypes (APMV1-9) indicated no relation to any of them. Next generation sequencing of whole genome sequences indicated the three isolates were genetically identical, and had a nucleotide structure typical for all APMVs, consisting of six genes 3’-NP-P-M-F-HN-L-5’. Phylogenetic analyses, and assessment of amino acid identities, suggested the most closely related lineages to be APMV-2, 8, 10 and 15, but the novel isolate had less than 64% identity to them and all other known avian paramyxoviruses. This value was above levels considered to generally define other APMV serotypes. Estimates of the evolutionary divergence of the nucleotide sequences of the genomes of APMVs have shown that novel Kazakhstan APMV strain was closest to APMV-2, APMV-8, APMV-10 and APMV-15, with calculated distance values of 2.057, 2.058, 2.026 and 2.286 respectively, which is above values considered to differentiate other serotypes (observed minimum was 1.108 between APMV-1 and recently isolated APMV/UPO216/Korea). Together, the data suggest that isolate APMV/gull/Kazakhstan/5976/2014 and other two should be considered as the first representative of a novel APMV-20 group, and is the first time that avian paramyxoviruses have been found infecting members of the gull family, extending the known taxonomic host range.

## Introduction

Avian paramyxoviruses (APMV) belong to the genus *Avulavirus* of *Paramyxoviridae* family possessing linear negative-strand RNA. According to the Taxonomy of the order Mononegavirales: update 2017 [1] they are divided into thirteen serotypes (APMV-1–13) based on a Hemagglutination Inhibition (HI) assay and comparison of genetic distances The most dangerous for poultry is APMV-1 (Newcastle disease virus), which is widespread around the world and causes significant economic damage. Other APMV serotypes APMV-2,3,5,7 are less pathogenic and cause respiratory infection or intestinal disease with varying degree of pathogenicity accompanied by a decrease in egg production, weight gain, conjunctivitis and pneumonia with variable mortality rates [2]. APMV-4, 8 and 9 have been isolated from ducks and other wild birds with no clinical signs of disease [3, 4]. APMV-6 virus causes mild respiratory disease and is associated with a drop in egg production in turkeys [5]. APMV-10-19 have been isolated exclusively from wild birds with no evidence of any disease.

The APMV genome encodes six major proteins: nucleocapsid protein (NP); phosphoprotein (P); matrix protein (M); fusion protein (F); hemagglutinin-neuraminidase (HN) and large RNA polymerase (L) as well as two non-structural proteins V and W [6]. APMV-6 has an additional SH gene, that is absent in other APMVs [7].

Very little is known about the molecular biology of APMV in wild bird populations, but an understanding of the molecular and pathological characteristics of APMV is of general epidemiological interest, and is important for developing vaccines in the case of emergence of novel pathogenic strains.

Until recently, the known APMV serotypes were restricted to APMV 2–9, which were isolated and characterised in the 1970s. Following expansion of viral surveillance initiatives and improvements in sequencing technology, ten novel serotypes have been discovered since 2001. A virus isolated from rockhopper penguins was antigenically and genetically distinct from all known serotypes 1–9 and considered to represent the prototype of a new group, APMV-10 [8]. A further novel APMV-11 isolate was obtained in France from a common snipe in 2010 [9]. APMV serotype 12 was isolated in Northern Italy in 2005 from a Eurasian widgeon [10]. Three recent publications have described a novel APMV-13 lineage independently found in three separate regions of Eurasia—Japan, Kazakhstan and Ukraine [11, 12, 13]. In 2017 six novel APMV serotypes were announced: from ducks in Japan [14] and Korea [15], from shorebird in Brazil [16] and three novel serotypes were simultaneously isolated from Antarctic penguins [17]. These data suggest that APMVs circulate widely in wild populations and that there is a high likelihood of novel genetically distinct variants emerging.

Kazakhstan has a vast territory crossed by large flyways, and hundreds of bird species concentrate in the natural landscapes during migration and breeding periods [18]. Five APMV serotypes including a novel APMV-13 have been isolated in Kazakhstan between 2003 and 2013 [19]. Here we present the results of the first isolation of a novel APMV from a gull sampled on the Caspian Sea coast in Kazakhstan, which represents a previously unknown serotype, and describe its antigenic, morphological and molecular characterization. To our knowledge this is the first isolation of avian paramyxovirus from gulls and we consider this serotype is specific for them.

## Materials and methods

### Ethical approval

All research components involving live animals were conducted according to regulations under the legislation ‘Rules for conducting biomedical experiments, preclinical (non-clinical) and clinical studies (№697, 12 November 2007, Republic of Kazakhstan)’, and were approved by the Institute of Microbiology and Virology Local Ethics Committee (Approval number: 2015-04-№1-ICPI).

### Sample collection

Cloacal and tracheal swabs were collected in 2013 and 2014 from live wild birds captured by ornithologists for banding during studies of migratory patterns. Sampling also was made during seasonal hunting for wild birds and from freshly dropped feces of wild aquatic birds in Northern Caspian seashore in Kazakhstan. For freshly dropped gull fecal samples it was not possible to identify the source species.

Samples were collected using sterile swabs which were subsequently placed in viral transport medium containing 2000 U/ml penicillin, 2 mg/ml streptomycin, 50 μg/ml gentamycin, 50 U/ml nystatin and 0.5% bovine serum albumin. In the field, samples were kept in liquid nitrogen, and were stored at -70°C after delivery to the laboratory.

### Virus isolation

Samples were first screened by RT-PCR targeting the M gene of avian influenza viruses (AIV). Then AIV-negative samples were inoculated into 10-day-old embryonated chicken eggs (ECE) and incubated 72 hours at +36°C [20]. The presence of virus in allantoic fluid was detected using Hemagglutination Assay with 0.75% chicken red blood cells.

### Virus cultivation

In order to define the virus growth characteristics, its replication was evaluated in two available in the laboratory cell lines: chicken embryo fibroblast (DF-1) and Madin Darby Canine Kidney (MDCK). Dulbecco’s minimum essential medium (DMEM) with 10% fetal bovine serum (FBS) was used and incubated at 37°C under 5% CO_2_. Monolayers with about 90% confluency were infected with 10^−3^ dilution of 2^8^ HA units of the virus with and without addition of trypsin (1 ug/ml) (Sigma). The cells were inspected every day for a week to evaluate cytopathic effects and hemagglutination activity of the cell culture supernatant.

### Intracerebral pathogenicity index (ICPI)

Virulence of this virus was defined by intracerebral introduction of virus-containing suspension into ten 1-day-old chickens (1:10 diluted, 50 ul) as described previously [21].

### Infection of 1-day-old and 4-week-old chickens

The pathogenicity of the virus was studied in one-day-old and 4-week-old non vaccinated chickens of Kobb 500 line inoculated intranasally. Chickens were inspected every day over one week for manifestation of any clinical symptoms of disease, and virus shedding was tested by PCR from cloacal and tracheal swabs.

### Production of rabbit antiserum

Antiserum to the novel APMV strain gull/Kazakhstan/5976/2014 was obtained by double immunization of rabbits with purified viral suspension. A first immunization was conducted by 15–20 intracutaneus injections near cervical and popliteal lymph nodes with antigen mixed with complete Freund’s adjuvant. After three weeks a second immunization was conducted intravenously with incomplete adjuvant. Blood was taken 7–14 days after second immunization [22].

### Hemagglutination Inhibition assay

Analysis was carried out using a standard HI assay [23], testing virus against antisera specific for the APMV 1–9 reference strains.

### Electron microscopy

Virus-containing allantoic fluids were centrifuged at 3000 RPM for 30 min. The supernatant was separated and then subjected to ultracentrifugation at 52500g for 40 min. The resulting pellets were diluted in a minimal volume of 0.05 M phosphate buffer. Virus specimens were prepared on formvar film-coated grids with the addition of a layer of evaporated carbon. The specimens were contrasted with 3% phosphotungstic acid solution and then examined in an electron microscope JEM 100 CX (JEOL, Japan).

### RNA extraction and RT-PCR

Viral RNA was extracted from 140 μl of hemagglutination assay positive allantoic fluid using the QIAamp RNA Mini kit (Qiagen, Hilden, Germany) in accordance with the manufacturer’s recommendations.

Reverse transcription PCR (RT-PCR) assays were performed on the basis of one-step protocol using appropriate RT-PCR kit (AccessQuick One-Step RT-PCR Kit, Promega) according to the manufacturer’s instructions, employing Pan-PMV primers targeting the conservative fragment of L-gene [24]. The cycling conditions consisted of 45 min at 48°C (reverse transcription) and then an initial denaturation at 94°C for 2 min followed by 35 cycles of 94°C for 15 s, 50°C for 30 s, and 72°C for 30 s and a final extension at 72°C for 10 min. The final PCR products were visualized on 2% agarose gel.

### Sequencing and phylogenetic analyses

After purification, the PCR products were Sanger-sequenced using the same PCR primers on an ABI 3730xl DNA analyser (Applied Biosystems, USA) using BigDye Terminator Kit v.3.1 Sequencing Kit (Applied Biosystems).

For whole-genome sequencing, viral RNA was used as a template for further library preparation using TruSeq Stranded Total RNA with Ribo-Zero Gold (Illumina, USA) according to manufacturer’s recommendations. Paired-end sequencing was performed on an Illumina HiSeq 3000 instrument. Raw sequence data were assembled and analyzed using CLC Assembly Cell software (Qiagen). Nucleotide and amino acid sequences were aligned and their evolutionary distances were estimated using Mega 6.0 software [25]. Twenty two other full-length APMV genome sequences from public databases were used as references for this analysis.

The most appropriate sequence evolution model for use in phylogenetic analyses was identified on the basis of Akaike Information Criterion (AIC) and Bayesian Information Criterion (BIC) ranking via ModelTest, as implemented in MEGA 6.0.

A maximum likelihood phylogenetic tree was constructed for concatenated whole genome nucleotide sequences, using a General Time Reversible model with discrete Gamma distribution and Invariant sites (2 category +*G*, parameter = 1.3410;+*I*, 13.6969% sites), with 500 bootstrap replicates, in MEGA 6.0 [26]. The tree is drawn to scale, with branch lengths measured in the number of substitutions per site. The analysis involved 22 APMV nucleotide sequences. Codon positions included were 1st+2nd+3rd+Noncoding. All positions containing gaps and missing data were eliminated. There were a total of 11538 positions in the final dataset.

Maximum-likelihood phylogenetic trees using amino acid sequences for the six individual APMV genes (NP, P, M, F, HN and L) were generated in MEGA 6.0, based on the Le_Gascuel_2008 model [27]. The phylogenetic trees are rooted at the midpoint. Numbers at nodes indicate maximum likelihood bootstrap values from 500 replicates under the specified model. Only values above 0.5 are shown.

### Nucleotide sequence accession number

The complete sequence of APMV/gull/Kazakhstan/5976/2014 is available at GenBank under the accession no. MF033136.

## Results

### Sample collection and virus isolation

**Fig 1:**
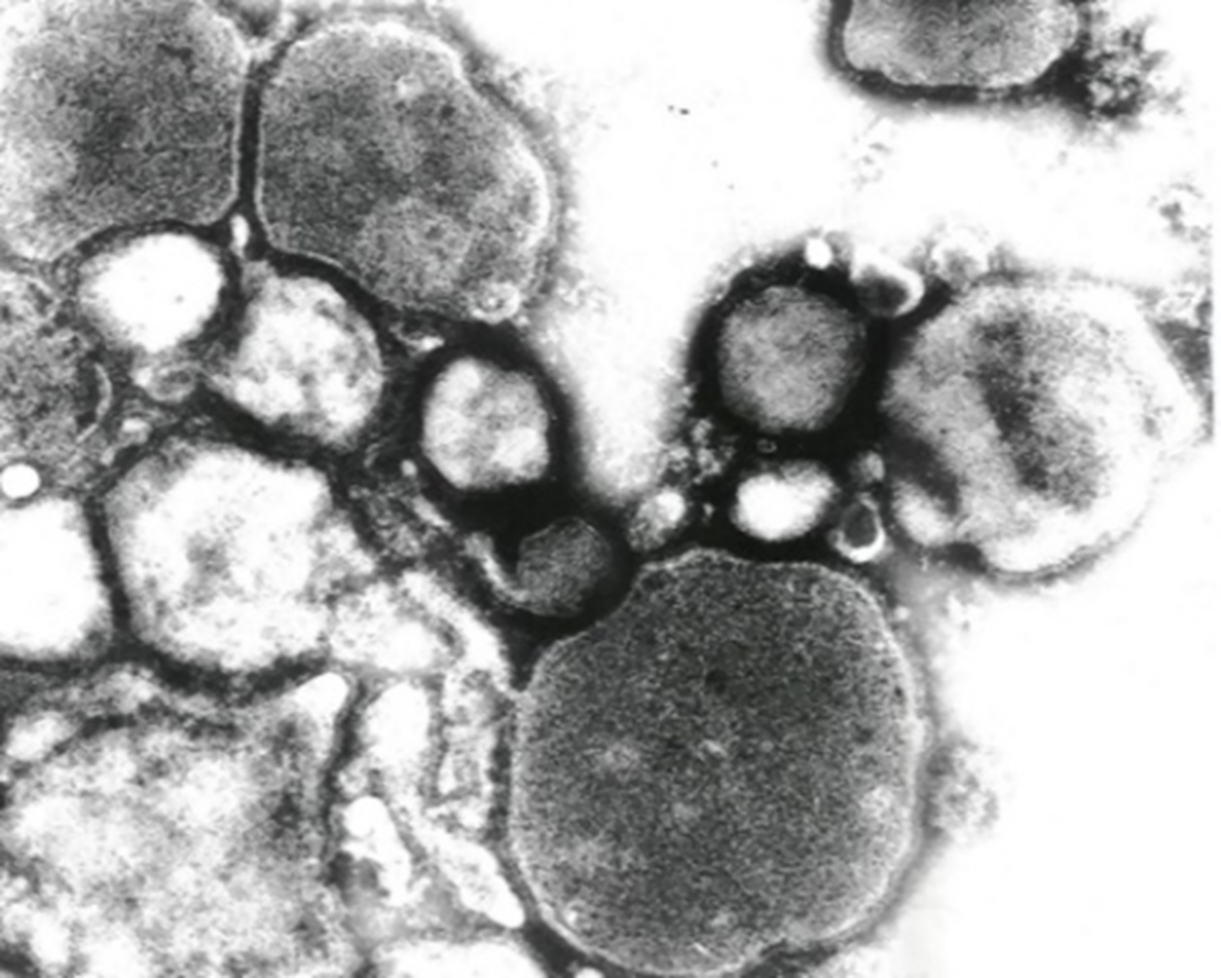
Electron microscopy morphology of negatively stained isolate APMV/gull/Kazakhstan/5976/2014. Magnification x 50000.

During annual virus surveillance, 486 samples were collected from 348 wild birds belonging to 50 species, and APMV positive samples were found in freshly dropped feces collected on the Caspian seashore in Kazakhstan. Samples were inoculated in ECE and after the first passage hemagglutinating agents were obtained. Viruses were isolated from 3 inoculums: APMV/gull/Kazakhstan/5976/2014, APMV/gull/Kazakhstan/5977/2014 and APMV/gull/Kazakhstan/5979/2014. Whole genome sequencing showed the viruses from the three independent samples to be 100% identical and so only APMV/gull/Kazakhstan/5976/2014 was used for further genetic analyses. Purified virus stock of APMV/gull/Kazakhstan/5976/2014 was examined by electron microscopy (Fig 1).

### Electron microscopy

The shape of virions had an expressed polymorphism. The size of spherical virions varied between 150–600 nm. The fine structure of virions was similar to other paramyxoviruses. Virions had 10–15 nm supercapsid envelope coated with 8–12 nm long spikes 4.5–5.0 nm in a diameter (Fig 1).

### Preliminary virus identification

The isolated hemagglutinating agents were tested using Hemagglutination Inhibition Assay and the Kazakhstan isolates did not react with reference sera specific to APMV-1 to APMV-9 indicating a different antigenic structure.

Kazakhstan isolates showed minimal cross-reaction with APMV-1, 2 and 6 indicating some antigenic similarity between these serotypes. The highest titers were registered with homologous antisera to this virus obtained from rabbits (Table 1).

**Table 1:** Hemagglutination Inhibition assay with antisera to reference APMVs and homologous rabbit antiserum.

Isolate	Immune sera to strains:											
	APMV-1/La Sota	APMV-2/Chicken/Yucaipa/56	APMV-3/Turkey/Wisconsin/68	APMV-3/Parakeet/Netherlands/449/75	APMV-3/Turkey/England/666/81	APMV-4/Duck/Hong Kong/D3/75	APMV-6/Duck/Hong Kong/199/77	APMV-7/Dove/Tennessee/4/75	APMV-8/Goose/Delaware/1053/76	APMV-9/Domestic duck/New York/22/78	APMV-13/white fronted goose/Northern Kazakhstan/5751/2013	APMV/gull/Kazakhstan/5976/2014
APMV/gull/Kazakhstan/5976/2014	20	20	0	0	0	0	20	0	0	0	0	**320**
APMV/gull/Kazakhstan/5977/2014	20	20	0	0	0	0	20	0	0	0	0	**320**
APMV/gull/Kazakhstan/5979/2014	20	40	0	0	0	0	40	0	0	0	0	**320**

Further identification of the serotype was made by Sanger sequencing of a 700 bp PCR product of L-gene fragment. A BLAST-analysis confirmed that the isolated virus sequence grouped within the APMV family, but did not match any currently known APMV.

### Growth characteristics of novel APMV strain

Avian paramyxovirus strain APMV/gull/Kazakhstan/5976/2014 yielded a titer of 2^8^ HA units in ECE, after 3 days incubation at 35°C. However, the virus did not replicate with and without exogenous protease in tested MDCK and DF-1cell lines that were used to determine whether they can support the growth of the virus.

The cells were inspected every day during a week and no cytopathic effect and hemagglutination activity of the cell culture supernatant were observed. Additionally PCR was run with supernatant and negative results were obtained, indicating that these cells did not support the replication of the novel gull APMV.

### Intracerebral pathogenicity index (ICPI)

After inoculation of the virus into the cerebrum of one-day-old chickens, the obtained ICPI value was zero, without any clinical signs of disease.

### Infection of 1-day-old and 4-week-old chickens

During every day observation of infected intranasally chickens, no manifestation of clinical symptoms of any disease was observed. All infected chickens looked healthy without any signs of sickness.

To reveal possible virus shedding polymerase chain reaction with cloacal and tracheal swabs was conducted. All tested chickens showed negative result indicating absence of virus infection.

### Sequence analysis of the whole genome

**Fig 2:**
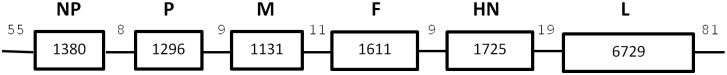
Schematic diagram showing the genomic organization of novel APMV.

The complete genome sequence of the APMV isolate was characterized using a ‘next-generation sequencing’ approach. Assembly of the reads produced a contig of 15,786 nucleotides, with a GC content of 42%, which is longer that of the genetically closest strains, APMV-2/Yucaipa (14904 bp), APMV-8/Delaware (15342 bp), APMV-10/Falkland Islands (15456) and APMV/Brazil (14952 bp). Review of the annotated genome identified the six genes typical for APMVs: 3’-NP-P-M-F-HN-L-5’ (Fig 2), which encode six viral proteins; NP– 459 amino acids (aa); P– 431 aa; M– 376 aa; F– 536 aa; HN– 574 aa, and L– 2242 aa. Therefore the genome of virus under study at 15,786 nucleotides follows the “rule of six”, characteristic for the most of APMVs.

Estimates of the evolutionary divergence of the nucleotide sequences of the genomes of APMVs showed that the novel Kazakhstan APMV strain was also closest to APMV-2, APMV-8, APMV-10 and APMV-15, with values of 2.057, 2.058, 2.026 and 2.286, respectively, compared to 1.108 (lowest) between APMV 1 and recently isolated APMV/UPO216/Korea, and 5.001 (highest) between APMV-4 and 8 (Table 2).

**Table 2:** Estimates of evolutionary divergence at the nucleotide level between all APMV serotypes sequences.

APMV Serotype	APMV 1	APMV 2	APMV 3	APMV 4	APMV 4	APMV 5	APMV 6	APMV 6	APMV 7	APMV 8	APMV 8	APMV 9	APMV 10	APMV11	APMV12	APMV13	APMV14	APMV Brazil	APMV Korea	APMV Antarctic A	APMV Antarctic B	APMV Antarctic C
APMV-1/mallard/US(MN)/99-376/1999																						
APMV-2/Chicken/California/Yucaipa/56	4,346																					
APMV-3/PKT/Netherland/449/75	4,724	4,645																				
APMV-4/duck/Hongkong/D3/75	4,745	4,721	3,975																			
APMV-4/KR/YJ/06	4,736	4,764	3,981	0,115																		
APMV-5/budgerigar/Kunitachi/74	4,353	3,533	4,663	4,710	4,703																	
APMV-6/Goose/FarEast/4440/2003	4,745	3,839	4,888	4,955	4,978	2,415																
APMV-6/duck/Taiwan/Y1/98	4,736	3,845	4,873	4,956	4,973	2,408	0,021															
APMV-7/dove/Tennessee/4/75	4,473	3,491	4,719	4,794	4,815	3,610	3,925	3,932														
APMV-8/Goose/Delaware/1053/76	4,376	1,863	4,645	4,764	4,745	3,542	3,991	4,001	3,382													
APMV-8/pintail/Wakuya/20/78	4,374	1,859	4,643	4,760	4,741	3,539	3,988	3,998	3,380	0,001												
APMV-9/duck/New_York/22/1978	1,739	4,388	4,801	**5,001**	4,987	4,497	4,804	4,813	4,423	4,379	4,376											
APMV-10/penguin/Falkland_Islands/324/2007	4,328	1,942	4,740	4,835	4,859	3,530	3,831	3,831	3,457	1,993	1,994	4,363										
APMV-11/common_snipe/France/100212/2010	4,362	3,324	4,592	4,730	4,697	3,330	3,898	3,901	3,447	3,380	3,380	4,455	3,377									
APMV-12/Wigeon/Italy/3920_1/2005	2,243	4,333	4,833	4,807	4,808	4,363	4,765	4,765	4,477	4,423	4,419	2,463	4,469	4,378								
APMV-13/goose/Kazakhstan/5751/2013	2,306	4,484	4,662	4,873	4,901	4,398	4,678	4,679	4,458	4,442	4,437	2,581	4,500	4,477	1,500							
APMV-14/duck/Japan/11OG0352/2011	4,469	3,647	4,691	4,884	4,874	2,924	3,371	3,379	3,691	3,452	3,448	4,580	3,525	3,615	4,496	4,448						
APMV/calidris_fuscicollis/Brazil/RS-1177/12	4,422	2,093	4,712	4,813	4,809	3,562	3,902	3,937	3,548	2,077	2,075	4,471	2,028	3,365	4,369	4,501	3,492					
APMV//WB/Korea/UPO216/2014	**1,108**	4,442	4,854	4,845	4,898	4,466	4,784	4,796	4,383	4,395	4,389	1,843	4,489	4,392	2,349	2,317	4,420	4,454				
APMV/Antarctic_penguin_virus_A	2,850	4,279	4,728	4,687	4,681	4,407	4,782	4,780	4,367	4,348	4,351	3,073	4,418	4,394	2,846	2,957	4,456	4,250	2,935			
APMV/Antarctic_penguin_virus_B	2,821	4,344	4,605	4,640	4,667	4,290	4,729	4,736	4,432	4,314	4,315	2,972	4,321	4,346	2,870	2,962	4,343	4,302	2,850	1,165		
APMV/Antarctic_penguin_virus_C	2,803	4,369	4,698	4,689	4,672	4,331	4,778	4,791	4,518	4,342	4,339	3,041	4,448	4,448	2,938	2,890	4,365	4,361	2,868	1,703	1,643	
APMV/gull/Kazakhstan/5976/2014	4,617	**2,057**	4,537	4,728	4,714	3,463	3,914	3,926	3,490	2,061	**2,058**	4,358	**2,026**	3,391	4,382	4,476	3,547	**2,286**	4,486	4,447	4,280	4,435

Bold values are distances between APMV-2, 8, 10, 15 and novel gull APMV; Bold values in shadowed cells are maximum and minimal interserotype distances. The number of base substitutions per site from between sequences are shown. Analyses were conducted using the Maximum Composite Likelihood model [28]. The rate variation among sites was modeled with a gamma distribution (shape parameter = 2). The differences in the composition bias among sequences were considered in evolutionary comparisons [29]. The analysis involved 23 nucleotide sequences. Codon positions included were 1st+2nd+3rd+Noncoding. All ambiguous positions were removed for each sequence pair. There were a total of 12916 positions in the final dataset. Evolutionary analyses were conducted in MEGA 6.

Calculation of the amino acid sequence identities of F and HN genes (Table 3) showed that novel Kazakhstan APMV shares a maximum identity of 62.5% and 53.48% with APMV-10/penguin/Falkland Islands/324/2007, 61.94% and 52.61% with APMV-2/Chicken/California/ Yucaipa/1956, 63.43% and 52.09% with APMV-8/Goose/Delaware/1053/1976 and finally 57.08% and 46.86% with APMV-15/calidris_fuscicollis/Brazil/RS-1177/12 respectively. The identities with other groups varied from 29.47% to 48.32% by F gene and from 31.7% to 43.37% by HN gene.

**Table 3:** Percent amino acid sequence identities in F and HN genes of avian paramyxoviruses representing all known APMV groups.

APMV Serotype	Gene	APMV 1	APMV 2	APMV 3	APMV 4	APMV 5	APMV 6	APMV 7	APMV 8	APMV 9	APMV 10	APMV11	APMV12	APMV13	APMV14	APMV Brazil	APMV Korea	APMV Antarctic A	APMV Antarctic B	APMV Antarctic C
APMV-1/La Sota	F	100																		
	HN	100																		
APMV-2/Chicken/California/Yucaipa/56	F	40.11	100																	
	HN	34.48	100																	
APMV-3/PKT/Netherland/449/75	F	31.3	29.85	100																
	HN	33.44	30.67	100																
APMV-4/duck/Hongkong/D3/75	F	31.82	33.95	32.22	100															
	HN	33.39	31.1	39.71	100															
APMV-5/budgerigar/Kunitachi/74	F	39.88	46.82	30.2	33.27	100														
	HN	33.79	42.5	32.05	29.87	100														
APMV-6/duck/HongKong/18/199/77	F	37.79	49.62	31.12	32.97	54.04	100													
	HN	30.67	41.03	30.84	31.1	56.62	100													
APMV-7/dove/Tennessee/4/75	F	37.47	38.24	***27*.*08***	29.49	37.1	37.1	100												
	HN	34.79	42.35	32.68	34.44	42.7	43.58	100												
APMV-8/Goose/Delaware/1053/76	F	40.51	63.43	31.12	34.99	45.11	48.8	38.58	100											
	HN	33.44	47.83	30.84	32.51	41.11	38.99	41.47	100											
APMV-9/duck/New_York/22/1978	F	54.8	37.87	28.54	28.49	36.39	36.84	34.13	37.38	100										
	HN	61.17	32.29	34.83	34.44	31.35	30.39	33.74	33.79	100										
APMV-10/penguin/Falkland_Islands/324/2007	F	40.11	61.38	28.73	33.02	46.82	49.81	37.68	61.00	39.17	100									
	HN	33.73	51.13	31.82	30.22	41.98	40.69	40.24	50.26	33.73	100									
APMV-11/common_snipe/France/100212/2010	F	33.99	42.16	30.57	32.02	39.33	39.63	35.43	40.69	31.94	41.04	100								
	HN	35,00	40.68	32.75	35.5	43.03	42.19	42,00	39.68	33.85	40.69	100								
APMV-12/Wigeon/Italy/3920_1/2005	F	54.57	37.87	29.65	32.05	37.5	37.91	34.87	36.09	51.83	38.05	32.96	100							
	HN	55.97	32.58	34.83	34.09	32.22	30.34	33.39	32.06	57.51	35.3	34.3	100							
APMV-13/goose/Kazakhstan/5751/2013	F	53.94	36.94	29.28	33.57	38.97	37.43	35.43	35.91	48.8	38.05	32.84	66.97	100						
	HN	54.07	33.33	36.04	34.97	32.4	31.6	36.2	33.62	54.4	33.73	35.75	62.34	100						
APMV-14/duck/Japan/11OG0352/2011	F	39.0	46.08	30.12	32.71	48.24	52.49	39.51	44.91	34.19	44.02	39.37	37.89	36.96	100					
	HN	34.14	41.89	34.66	32.51	52.43	51.03	43.05	41.76	33.16	40.34	44.13	34.48	34.71	100					
APMV-15/calidris_fuscicollis/Brazil/RS-1177/12	F	38.92	58.2	30.25	32.47	46.3	47.23	37.29	57.19	36.9	58.95	43.35	37.63	37.82	45.1	100				
	HN	31.02	45.07	31.88	32.33	38.67	37.99	38.13	47.83	31.43	45.56	37.99	32.29	31.95	39.37	100				
APMV//WB/Korea/UPO216/2014	F	**73.68**	41.23	30.57	32.12	38.41	38.11	35.43	40.69	55.35	41.41	34.48	53.11	53.94	39.37	38.19	100			
	HN	***70*.*71***	34.13	33.44	34.79	32.57	***27*.*89***	35.67	35.52	62.17	33.73	33.44	54.56	58.03	33.79	32.29	100			
APMV/Antarctic_penguin_virus_A	F	46.76	37.68	28.09	33.45	38.26	36.59	35.62	36.59	43.43	37.12	34.01	40.66	42.14	36.04	37.7	48.05	100		
	HN	47.48	34.65	37.6	34.79	33.27	30.55	37.43	36.22	46.45	36.34	33.61	47.41	48.35	35.51	34.36	45.4	100		
APMV/Antarctic_penguin_virus_B	F	46.04	37.31	31.3	33.51	35.35	38.67	34.69	36.83	42.72	37.12	33.88	42.35	41.43	38.63	37.26	47.14	64.32	100	
	HN	46.27	33.62	34.83	36.2	31.35	28.93	34.97	34.31	45.42	33.73	32.41	46.19	48.87	33.1	32.46	44.16	65.82	100	
APMV/Antarctic_penguin_virus_C	F	44.84	36.56	28.91	31.09	37.5	34.95	33.76	36.27	43.73	38.24	32.2	43.22	44.4	36.04	37.26	45.91	57.48	55.06	100
	HN	46.96	30.86	35.52	36.37	32.75	30.32	34.44	33.96	48.35	34.6	32.07	48.55	49.22	33.96	32.46	47.7	51.27	51.78	100
APMV/gull/Kazakhstan/5976/2014	F	39.92	**61.94**	29.47	33.76	47.76	48.32	36.94	**63.43**	38.61	**62.5**	43.47	36.75	36.94	44.02	**57.08**	41.04	38.43	38.24	37.87
	HN	34.32	**52.61**	31.7	32.33	42.85	40.94	40.24	**52.09**	35.36	**53.48**	40.59	34.49	34.66	43.37	**46.86**	36.23	37.63	35.19	34.49

Bold values are distances between APMV-2, 8, 10, 15 and novel Kazakhstan APMV; Bold values in italics are maximum and minimal interserotype distances. Identity = 100 x number of nucleotide matches/total number of nucleotides. BLOSUM62 Matrix: BLOSUM Clustered Scoring Matrix in 1/2 Bit Units. Cluster Percentage: > = 62, Entropy = 0.6979, Expected = -0.5209

The putative amino acid sequence of the F-gene cleavage site was defined as GEQQAR↓LIG. This motif lacks multiple basic residues, a characteristic that typically corresponds with non-pathogenic variants, which is concordant with results of ICPI test.

### Phylogenetic analyses of the complete genomes and separate amino acid sequences of all genes of published APMV serotypes

**Fig 3:**
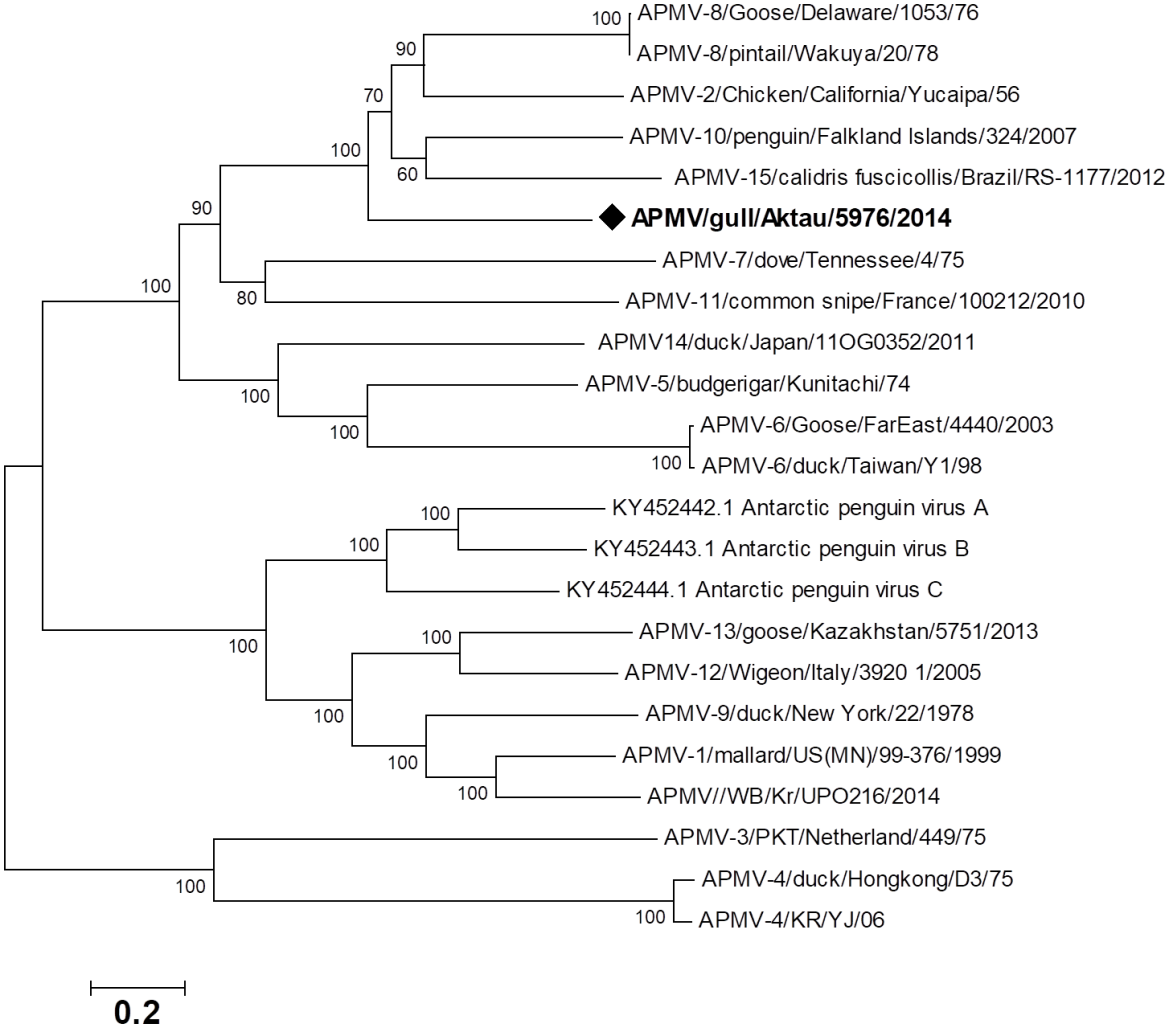
Phylogenetic relationships on nucleotide level between novel APMV serotype and others.

A maximum likelihood phylogeny of the whole genome nucleotide sequence places the novel gull APMV in a clade with serotypes APMV-2, 8, 10 and 15, lying on a separate branch basal to this group (Fig 3).

**Fig 4:**
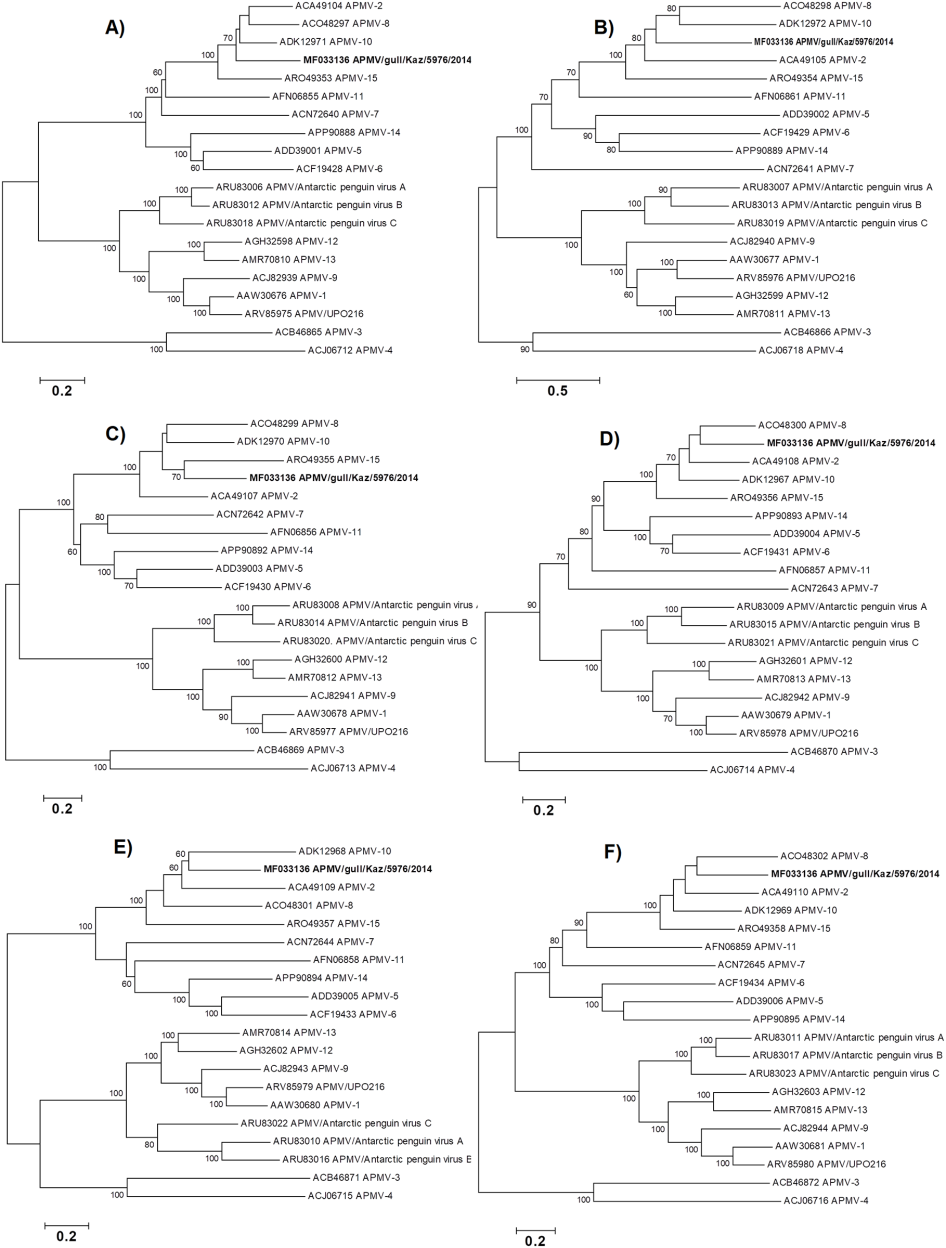
Phylogenetic analysis of the novel APMV/ gull/Kazakhstan/5976/2014 proteins in comparison to other serotype APMV is shown as follows: A) nucleocapsid protein, B) phosphoprotein, C) matrix protein, D) fusion protein, E) hemagglutinin-neuraminidase protein, and F) large polymerase protein.

The close evolutionary relationship with APMV-2, APMV-8, APMV-10 and APMV-15 is also supported the analyses of the 6 individual APMV genes at the amino acid. (Fig 4).

## Discussion

Kazakhstan has a vast territory where three large flyways of water birds cross: Black Sea-Mediterranean, West Asian-East African and Central Asian. Numerous places of nesting, moulting and summer concentration of birds play an important role in the maintenance of the populations of more than 150 water bird species inhabiting Central Asia.

Historically, five different APMV serotypes have been isolated in Kazakhstan between 2002 and 2013. APMV-1 serotype was isolated in 2005, 2006 and 2009 from wild birds in Central Kazakhstan and in 2013 from avifauna of Southern Kazakhstan. APMV-4 from wild birds was isolated in 2004 and 2009 in South-eastern and Caspian regions. APMV- 6 and 8 were first isolated in Southern and Northern Kazakhstan respectively in 2013. Aside from these, an APMV-2 strain was also isolated during the 1980s from chickens and turkeys in Kazakhstan [30]. Recently a novel serotype 13 was also isolated in Central Kazakhstan. Thus the circulation of a variety of APMV among wild bird species in this region is evident.

This study found APMV positive samples during annual screening of gulls from the Caspian seashore in Kazakhstan. The electron microscopy showed the isolated virus to have morphology consistent with paramyxoviruses but it was not possible to differentiate between APMV serotypes on this basis.

Traditionally, APMVs were resolved based on their antigenic differences, and nine groups were defined by Hemagglutination Inhibition Assay in the 1970s. Since the isolation of APMV-10, it was suggested to classify APMVs based on genetic data because of frequent cross-reactions in HI assays, and a possible lack of hemagglutinating activity in potential future serotypes, as observed in APMV-5 [31].

In this research the Kazakhstan isolate, as expected, showed the highest titers with homologous antisera, but also minimal cross-reaction was registered with APMV-1, 2 and 6, confirming the priority of the genetic approach.

The novel APMV did not cause death of chicken after intracerebral inoculation with 2^8^ HA units of virus, indicating that it is avirulent for them. Experimental intranasal infection of 1-day-old and 4-week-old chicken by virus also the virus was avirulent in chickens.

The virus did not replicate with and without exogenous protease in tested MDCK and DF-1 cell lines, however, the growth characteristics needs to be evaluated with other cell lines.

Sequencing of the viral genome revealed characteristic APMV coding regions and the noncoding terminal sequences (e.g. 55 nucleotide non-coding leader sequence at the 5’ end that is present in all APMV). The genome size is 15,786 nucleotides that is compatible with the rule of six and it also has 6 genes typical for APMV: 3’-NP-P-M-F-HN-L-5’.

The phylogenetic relationship among APMV-2, APMV-8, APMV-10 and APMV-15 is consistent throughout the genome, at both the amino acid and nucleotide level, forming a monophyletic group, suggesting these viruses share a more recent common ancestor than with other lineages. Their close relatedness observed in phylogenetic trees was also reflected in the pattern of nucleotide sequence identities. While there is no unified criterion for differentiating APMV serotypes based on evolutionary divergence, the distance separating novel APMV from the closest lineage, APMV-2 is 2.057, and this divergence is greater than the minimum inter-serotype distances separating other accepted serotypes (minimum 1.108). This, together with the other serological data, suggests that isolated new APMV should be considered as a novel serotype and the voucher strain of a new APMV-20 group [32].

Finally, to our knowledge this is the first report of an APMV in gulls (avian family *Laridae*). The evolutionary divergence of the virus suggests the serotype could be specific for gulls, and it may be persisting in the population,.
